# Perceptions of Parental Needs in General Pediatric Inpatient Units: A Comparative Study Between Nurses and Parents in Saudi Arabia

**DOI:** 10.3390/children12070947

**Published:** 2025-07-18

**Authors:** Hawa Alabdulaziz, Malak Alharthi, Sara Alhazmi, Alyaa Hawsawi, Shahad Almuhyawi, Zahra Almalki

**Affiliations:** 1 Faculty of Nursing, King Abdulaziz University, P.O. Box 24828, Jeddah 21551, Saudi Arabia; maoalharte@kau.edu.sa (M.A.); salhazmi0096@stu.kau.edu.sa (S.A.); aishaghassan@stu.kau.edu.sa (A.H.); 2 Dr. Suliman Al-Habib Hospital, P.O. Box 8929, Jeddah 22245, Saudi Arabia; sawadalmahiawi@stu.kau.edu.sa; 3 Maternity and Children’s Hospital, Ministry of Health, P.O. Box 13877, Jeddah 21414, Saudi Arabia; zaaaalmalki@moh.gov.sa

**Keywords:** hospitalized children, need of parents, family-centered care, Saudi Arabia, pediatric nurses

## Abstract

**Introduction**: Hospitalization of children creates significant emotional and psychological stress for parents, highlighting the importance of addressing their needs in pediatric care settings. **Aims**: This study examines the perceptions of both parents and pediatric nurses regarding the needs of hospitalized children. **Method**: A cross-sectional survey using the validated Needs of Parents of Hospitalized Children (NPQ) was administered to 218 parents and 218 pediatric nurses in four hospitals in Jeddah, Saudi Arabia. Key domains assessed included trust, information, and support. Group differences were evaluated using non-parametric statistical analyses. **Results**: Trust was prioritized more by parents (83.9%) than nurses (72.4%) (*p* < 0.05). Both groups deemed information important, but parents (87.2%) rated it as more necessary than nurses (74.1%) (*p* = 0.02). Parents (79.8%) expressed a greater need for support compared to nurses (67.3%) (*p* = 0.03). **Conclusions**: This study identified perceptual differences between parents and nurses regarding trust, communication, and support. Some differences were statistically significant at the *p* < 0.01 level, while others were suggestive (*p*-value between 0.01 and 0.05) and require further investigation. These disparities suggest a need to foster mutual understanding and improve communication practices to better align healthcare delivery with family expectations and strengthen family-centered care.

## 1. Introduction

Family-centered care (FCC) refers to an approach to healthcare that recognizes families as essential partners in the care process, particularly for children requiring ongoing support [1,2,3]. The FCC principles highlight the need for parent or family participation in the provision of care because they are the primary caregivers and decision-makers [3]. Families have a crucial role to play in the health and well-being of children, particularly in their outcome [1]. These principles—understanding the family’s perspective, information exchange, coordination of care, and involvement of families and healthcare workers—are expected to establish an environment that addresses the emotional, informational resources, and practical issues for not only the child but also for the family [4,5]. As defined by the American Academy of Pediatrics (AAP), FCC “is an approach to the design, implementation, and assessment of healthcare that is based upon active collaboration between healthcare professionals, patients, and families” [6]. In pediatric settings, FCC is vital because children are frequently unable to express themselves due to their age, degree of development, or health status. Previous literature reported that FCC improves positive healthcare outcomes, increases satisfaction levels among families, and lowers stress levels for both children and parents [1,7].

In addition to established FCC principles, this study is grounded in the Fundamentals of Care (FoC) framework, which underscores the central role of therapeutic relationships, emotional support, and relational communication in nursing practice. Recent work in nursing education has highlighted FoC’s importance in structuring care curricula to develop person-centered nurses [8]. An evolutionary concept analysis also affirmed FoC’s emphasis on communication, contextual environment, and nurse–patient relationship [9]. Furthermore, European bedside nurses have endorsed FoC as a practical framework to articulate complex care tasks in real-world settings, aligning physical, psychosocial, and relational care domains [8,10]. In pediatric settings, where parental trust, information exchange, and emotional reassurance are paramount, FoC serves as a complementary lens to analyze how trust and communication contribute to family-centered care.

The World Health Organization (WHO) has been advocating for critical attention towards the incorporation of FCC, with a focus on enhancing patient care and the quality of healthcare services offered to children and other vulnerable groups [10]. Recently, several countries have renewed interest in accepting FCC as the best practice in pediatric care [2,3,8,9,10]. Recent evidence suggests that FCC-involved parents in the care of their hospitalized children strengthened their emotional bond, improved communication with healthcare staff, and built a stronger trust toward healthcare settings [3,5,11,12]. Likewise, research demonstrated that when healthcare providers collaborate with families effectively, it can also reduce anxiety for both parents and children [13]. These practices had been recommended and practiced in the one study in Australia [14] and one study in Turkish [15] healthcare systems, where family involvement is often seen as a central tenet of healthcare delivery.

In Saudi Arabia, family is widely regarded as the core unit of society, and the healthcare system reflects this strong cultural norm [16]. In recent decades, the country’s healthcare system has undergone significant transformation with substantial investments made in hospitals’ modernization, improving healthcare services and infrastructure [17]. The country boasts a mix of public and private healthcare providers, and most hospitals, including those in major cities, are well-equipped to handle complex medical procedures [17]. The Saudi healthcare system is primarily governed by the Ministry of Health, which oversees most public healthcare institutions [18]. There are also specialized hospitals that provide advanced medical care in various specialties, including pediatrics.

While these advancements, the healthcare system in Saudi Arabia still faces challenges related to the integration of family-centered care [2,19,20]. In the past, the healthcare approach has often been more paternalistic, with doctors and nurses making decisions on behalf of patients and families [21,22]. However, with ongoing reforms, there is increasing recognition of the importance of involving families in the care process, particularly in pediatric settings [23,24]. Nurses, as frontline healthcare providers, play a critical role in the delivery of FCC. They act as crucial intermediaries between families and doctors, guaranteeing the understanding and respect of the family. Nurses are responsible for providing direct clinical care to the patient, managing the patient’s family members emotionally, providing details, and fostering interaction between the family members and the healthcare team [15,16]. Despite the widespread acceptance of its significance, a significant gap persists between the perceptions of healthcare providers and parents about the fulfillment of parental needs.

Healthcare providers may believe that they are addressing the emotional and psychological needs of parents, while parents themselves may feel unsupported in these areas [14,24]. Various factors, such as differing understandings of emotional support and its delivery, contribute to this discrepancy. This suggests that there are much more complex issues regarding the needs of parents and how exactly these needs are addressed in clinical settings. This gap in the literature lays the groundwork for crucial research.

Therefore, this study aimed to assess and compare the perceptions of nurses and parents regarding the informational, emotional, and trust-related needs of families during pediatric hospitalization in Saudi Arabia. Specifically, it sought to (1) identify perceived differences in the importance and fulfillment of these needs between parents and nurses and (2) assess whether these perceptual differences suggest implications for family-centered care practices. We hypothesized that parents would rate these needs as more important and less adequately met compared to nurses. The findings of this study could contribute to improving family-centered care by providing insight into the unique needs and expectations of parents within this cultural and healthcare context.

## 2. Materials and Methods

### 2.1. Study Design and Setting

This study employed a cross-sectional descriptive design using a convenience sample of participants. Data were collected from four general pediatric inpatient units across four tertiary hospitals in Jeddah, Saudi Arabia. These units provide inpatient care for children with a range of non-critical medical and surgical conditions. This study focused specifically on routine hospitalization in order to compare parent and nurse perceptions regarding family needs in general pediatric care settings.

### 2.2. Study Participants

A convenience sample of parents of hospitalized children and pediatric nurses was recruited from general pediatric wards in a tertiary hospital. Inclusion criteria for parents were as follows: (1) having a child currently admitted to a general pediatric unit; (2) being the primary caregiver during hospitalization; and (3) ability to read and understand Arabic. Nurses were eligible if they provided direct care to pediatric inpatients and had at least one year of pediatric nursing experience. Exclusion criteria included parents of children with chronic illnesses or those hospitalized for more than two weeks, as this study aimed to capture perceptions in acute care settings. Parents of children admitted to the Neonatal Intensive Care Unit (NICU) and Pediatric Intensive Care Unit (PICU) were also excluded, as were nurses working exclusively in those units.

### 2.3. Sampling and Sample Size

Raosoft Sample Size Calculator (Raosoft Inc., Seattle, WA, USA) was utilized for sample size computation [25]. For the parent respondents, the total population consists of 316 parents of hospitalized children (95% confidence level and 5% margin of error; the required sample size was 174 parents). Similarly, the total population of pediatric nurses consisted of 270 nurses who were employed in pediatric units. Using the same margin of error and confidence level, the sample size was 159 for nurses. These sample sizes were sufficient to ensure the statistical power required for meaningful analysis and comparison of parents’ and nurses’ perceptions of hospitalized children’s needs. Participants were selected using convenience sampling.

### 2.4. Data Collection

Data were collected over a two-month period (January and February 2023) from pediatric nurses and parents of hospitalized children in four general pediatric units at tertiary hospitals in Jeddah, Saudi Arabia. After obtaining institutional approval and informed consent, participants completed the Needs of Parents Questionnaire (NPQ) in either English or Arabic, depending on their language preference. Data collection from parents took place while their child was still hospitalized, ensuring that responses reflected real-time perceptions rather than retrospective accounts. The surveys were distributed in paper format by trained research assistants during scheduled hospital visits or shift hours. Parents were approached in waiting or inpatient areas, while nurses were contacted during break periods to minimize disruption to clinical duties. Completed questionnaires were collected anonymously on-site and stored securely for analysis.

After using the tool, this study divided participants into the following two groups:

1. Pediatric Nurses: Participants who met the inclusion criteria received a hard copy of the questionnaire to complete during the focus group discussions.
2. Parents of Hospitalized Children: We provided a hard copy and/or an electronic copy of the NPQ to participating parents who met the inclusion criteria. Each participant was provided with a questionnaire, an information sheet about the research, and informed consent highlighting the purpose of the research, the voluntary nature, and the confidentiality of research participants.

Researchers obtained completed questionnaires and informed consents, then sealed them in a box to ensure confidentiality. The researchers observe ethical considerations at all stages of data collection and protect the information from identification.

### 2.5. Instruments

The “Needs of Parents of Hospitalized Children” (NPQ) questionnaire, developed by Kristjansdottir [21], was used in this study due to its comprehensive coverage of the various needs parents may experience when their child is hospitalized. The tool includes 51 items focused on assessing different dimensions of parents’ needs in the hospital setting. The NPQ evaluates six key domains: trust, to be trusted, information, support and guidance, human and physical resources, and family [23,26]. Each item is rated across the following three dimensions: importance (how important do you perceive the following statements are for you in relation to your child’s hospitalization?); fulfillment (the need, concern, or service presented in the statement—how well and to what extent do you feel it is being met?); and independence (is it your opinion that the hospital should help you to fulfill the particular needs that you have perceived from this statement?). A 3-point Likert scale was employed to evaluate both the importance and fulfillment of each item, with scores assigned as follows: 3 indicating “Very important/Fully met,” 2 indicating “Important/Partially met,” and 1 indicating “Not important/Not met.” Meanwhile, independence was measured with a binary response: 1 = Yes (help needed) and 2 = No (independent).

Given the linguistic diversity of the study population, the NPQ was initially used in its original English version with permission from the author, obtained via email correspondence. The questionnaire was then translated into Arabic by qualified pediatricians and supervisors familiar with the Saudi healthcare context. A back-translation process was employed to ensure semantic and conceptual equivalence between the English and Arabic versions. The final Arabic version was reviewed and approved by nursing educators and nursing supervisors to confirm its accuracy, cultural appropriateness, and relevance to the study objectives.

A small sample of parents and healthcare providers pilot-tested the translated Arabic version of the NPQ to evaluate its clarity, comprehensibility, and cultural appropriateness. Suggested modifications based on the responses from the pilot sample helped improve the tool. Feasibility studies like these aid in elucidating whether any vague terms or concepts that could skew the response patterns exist. This crucial phase in the research design stage ensures a thorough cross-cultural comparison while maintaining the tool’s validity across diverse languages [24]. Various invariants of structures or factors were identified in the basic psychometric studies of the NPQ and have been established in other populations. After the NPQ modification, Kristjánsdóttir’s original research in 2018 revealed an alpha measure estimate of 0.89, indicating a satisfactory score on the internal consistency measure. Expert judgment and factor analysis also verified the content and construct validity of the instrument. Reports indicate that countries such as Australia, Sweden, Canada, South Korea, and Brazil have been using the tool with consistent results across diverse settings [14].

Likewise, the reliability of the Arabic version of the NPQ was also assessed. The translated version maintained a high Cronbach’s alpha of 0.89, further confirming its reliability for use in the Saudi Arabian context. We closely monitored the translation process to ensure no loss or alteration of meaning occurred during the adaptation. Expert reviews from local pediatric nursing educators also supported the reliability, confirming that the translated version was culturally appropriate and retained the intended meaning of each item.

### 2.6. Ethical Considerations

The relevant institutions granted ethical approval for this study, ensuring compliance with ethical research standards. The Institutional Review Board (IRB) at the Faculty of Nursing in King Abdulaziz University granted ethics approval on the 5th of November 2022 (Ref No. 1F.07). The same goes for the Research Ethics Committee at King Abdulaziz University Hospital (Ref No. 3023; dated 15 January 2023) and the Ministry of Health MOH (Ref No. A01544; dated 9 January 2023). Both parents of hospitalized children and pediatric nurses were informed by written consent that the study was voluntary and that they could withdraw at any time. This study followed secure storage procedures and anonymized all collected data to ensure confidentiality. The researcher adhered to the principles of privacy and data protection, ensuring that participant information remained confidential at all stages of the research.

### 2.7. Data Analysis

This study utilized the Statistical Package for the Social Sciences (SPSS) software, version 26, a widely used tool for handling and analyzing quantitative data in research. Specifically, frequencies and percentages were used for such demographic profiles and the importance, fulfillment, and independence scores, based on parents’ and nurses’ perceptions of the needs of hospitalized children. The one-sample Kolmogorov–Smirnov test shows that the data for both the parents and nurses are not distributed normally. The overall Kolmogorov–Smirnov Z statistics are 0.36 for the parent group and 0.38 for the nurses (*p* < 0.001). Given the non-normal distribution of scores, Mann–Whitney U tests were used to compare NPQ domain scores between groups. Chi-square tests were applied for categorical comparisons.

Given the number of individual item comparisons conducted, the potential for Type I error inflation due to multiple testing was acknowledged. While a formal multiplicity correction (e.g., Bonferroni adjustment) was not applied due to the exploratory nature of the analysis, results were interpreted conservatively. Given the number of comparisons, we adopted a conservative threshold of *p* < 0.01 for statistical significance. Moreover, *p*-values between 0.01 and 0.05 were interpreted as suggestive trends, not statistically significant.

## 3. Results

A total of 218 parents and 218 nurses completed the Needs of Parents of Hospitalized Children (NPQ) survey.

### 3.1. Parent Demographics

Table 1 shows that the majority of parents in this study were mothers, comprising 199 participants (91.3%); 107 (49.1%) were aged 31–40 years, and 87 (39.9%) were university graduates. The children of the participants were predominantly aged 1–5 years (83 children, 38.1%), 137 (62.8%) had a history of previous hospital admissions, and 137 (62.8%) had previous hospital stays lasting less than 10 days. Most parents (154, 70.6%) were not employed.

### 3.2. Nurses Demographics

Among the nurses, 112 (51.4%) were aged 31–40 years, 142 (65.1%) were non-Saudi nationals, and 114 (52.3%) had university-level qualifications. The nurses’ years of experience ranged from 1 to 18 years, with a mean (SD) of 5.34 (2.78) years (see Table 1).

This study compared parent and nurse perceptions across six key domains of pediatric care using NPQ mean scores: trust, being trusted, information, support and guidance, human and physical resources, and family-centered care (see Table 2). Parents strongly valued trust in the healthcare setting, placing a higher priority on trust-based interactions with nurses than nurses perceived. This was reflected in a suggestive difference in importance (*p* = 0.01) and fulfillment (*p* < 0.001) (see Table S1). Similarly, in the domain of being trusted, a trend-level difference in fulfillment was found (*p* = 0.02), suggesting that parents felt less trusted by nurses than nurses believed (see Table S2). For informational needs, both groups agreed on its importance (*p* = 0.29) and on the adequacy of current delivery (*p* = 0.60). However, a significant gap in independence (*p* = 0.02) revealed that parents felt more reliant on nurses for access to information, underscoring their stronger desire for clear, ongoing communication (see Table S3). In terms of support and guidance, both groups aligned with importance (*p* = 0.18) and fulfillment (*p* = 0.16), yet a trend-level difference was present in independence scores (*p* = 0.03), not meeting the *p* < 0.01 criterion for statistical significance (see Table S4). No significant differences were found in the domains of human and physical resources (see Table S5) or family-centered care (see Table S6), with parents and nurses reporting similar views on importance, fulfillment, and independence.

## 4. Discussion

This study examined the perceptions of both nurses and parents regarding the needs of hospitalized children’s parents. The investigation yielded several findings.

The findings indicated that parents perceived trust as significantly more important, more frequently fulfilled, and more essential to their care experience than did nurses. While prior studies have shown that healthcare providers may underestimate the level of trust parents require [7,12,27], our findings add to this literature by demonstrating that such perceptual gaps are also evident in pediatric settings within this study’s context. In environments where a child’s vulnerability increases parental emotional stress, trust may hold distinct meaning for parents. These findings align with the Fundamentals of Care (FoC) framework, which emphasizes trust as a core element of relational care between healthcare providers and patients or families. According to the FoC framework, establishing and maintaining trust is central to effectively meeting patients’ emotional and informational needs. The observed divergence in perceptions between nurses and parents may signal missed opportunities for relational engagement, particularly in high-stress environments such as pediatric wards. Although the current study does not explore the underlying causes of this divergence, it underscores the importance of further inquiry into how trust is constructed, enacted, and sustained within culturally specific pediatric care contexts. As Adolfo et al. [28] note, trust influences not only communication but also overall satisfaction, suggesting that addressing relational gaps could enhance the quality of family-centered care. Whether these perceptual discrepancies are shaped by systemic, cultural, or organizational factors remains a topic for future research.

Second, results revealed that both parents and nurses agreed on the importance of parents feeling trusted. However, nurses perceived a higher frequency of meeting this need compared to parents. This disparity suggests that while nurses believe they are adequately addressing the need for trust, parents may feel otherwise [3,27]. Parents expressed a higher level of unmet need for acknowledgment of their trust, an important finding. This highlights a relational dynamic between parents and nurses, emphasizing the necessity for more effective strategies to foster mutual trust. This result aligns with the findings of Alahmari et al. [19], who reported that healthcare providers frequently overestimate the extent to which they meet the emotional and relational needs of parents in pediatric settings. Similarly, Davies et al. [29] found that parents often perceive clinical interactions as neglecting their emotional needs, particularly the need for trust. In contrast, the findings diverge from those of Cruz et al. [30], who argued that well-structured communication protocols can reduce such perceptual gaps, suggesting that institutional or cultural factors may influence how trust is experienced and interpreted. While nurses may focus on providing accurate information or providing professional assurance, parents tend to associate trust with inclusion in decision-making or empathetic listening to their problems [30]. These findings reinforce the need to further investigate how different stakeholders conceptualize and evaluate relational components of care within pediatric settings.

Third, another significant finding revealed that both parents and nurses agreed on the importance of informational needs, but parents placed greater emphasis on the need for information than nurses did. Interestingly, both groups reported a similar perception regarding the extent to which these informational needs were met. This perceptual difference may reflect variations in expectations or communication styles, rather than actual deficiencies in care delivery. These findings align with previous research demonstrating the fundamental importance of communication in pediatric care, as parents frequently seek updates and explanations about their children’s health status [31,32]. A discrepancy in perceived importance may increase the risk of underestimating the informational expectations of parents. Moreover, while informational needs were met to a comparable extent, it is possible that parents’ heightened valuation of these needs relates to concurrent emotional stress associated with their child’s hospitalization—an area warranting further exploration. Similarly, findings showed that both parents and nurses regarded guidance and support as important, yet parents reported a higher perceived need for support compared to nurses. This difference reinforces the broader theme of perceptual divergence and highlights the value of exploring how emotional and informational needs are interrelated in the context of family-centered pediatric care.

Finally, both parents and nurses perceived the need for support and guidance as important and reported that it was generally being met. However, parents rated this need as more urgent than did nurses, indicating a perceptual difference in the prioritization of support-related aspects of care. This finding aligns with previous research showing that healthcare providers may overestimate the extent to which they fulfill parents’ emotional and practical support needs, particularly during stressful periods of hospitalization [4,5,31]. For example, some studies indicate that parents of children admitted to the hospital urge more emotional support along with information, particularly in emergencies [14,33,34]. The heightened emotional investment that parents experience during their child’s hospitalization may influence their perception of the urgency and adequacy of support. These observations are supported by the FOC framework, which identifies the nurse–patient relationship as central to the effective delivery of care. Within the FoC model, the relational domain—including trust, presence, and communication—acts as the foundation through which emotional and psychosocial needs are recognized and addressed. The perceptual divergence between parents and nurses highlighted in this study may reflect inconsistencies in how these relational elements are enacted in clinical practice.

While this study does not explore the underlying causes of these perceptual differences, it underscores the importance of further research into how support is understood and evaluated by both families and healthcare professionals within pediatric contexts. To the best of our knowledge, there is a paucity of empirical research examining perceptual differences between parents and nurses regarding trust, communication, and support within pediatric inpatient settings in Saudi Arabia or the broader Gulf region. The current findings contribute to the growing body of literature on family-centered care by elucidating areas of perceptual divergence that warrant further exploration. These insights may be of particular relevance in informing culturally responsive care models and guiding future region-specific investigations.

These perceptual differences between parents and nurses regarding trust, communication, and emotional support have potential implications for family-centered care. While this study does not evaluate clinical outcomes or the impact of specific interventions, the findings point to areas where expectations between care providers and families may diverge. Previous research suggests that misalignment in perceptions of support or communication can affect parental satisfaction and engagement in care [35,36]. Addressing such divergences may require further research into how trust and information are operationalized in everyday pediatric practice and whether cultural, institutional, or role-based factors contribute to these differing viewpoints. Understanding these gaps more deeply could inform the development of contextually appropriate strategies for enhancing parent–nurse collaboration and optimizing family-centered care in pediatric settings.

### 4.1. Limitation of This Study

Some limitations should be considered when interpreting the findings. Firstly, the use of convenience sampling may limit the generalizability of findings. Additionally, the majority of the parents in this study were mothers (86.6%), which may introduce gender bias and affect the broader result’s applicability. The exclusion of children with chronic illnesses or prolonged hospital stays further narrows the relevance of the findings to more acute care settings. Moreover, data were collected from four hospitals in one city and one region of Saudi Arabia, which may limit the generalizability of the findings to broader populations or healthcare settings beyond the studied hospitals. From a methodological standpoint, the cross-sectional design precludes causal inference and provides a lower level of evidence compared to longitudinal or mixed-methods designs. Additionally, this study did not collect or report diagnostic data regarding the children’s specific medical conditions at the time of hospitalization. This limited our ability to analyze how the type or severity of illness may have influenced parental perceptions or needs. Future studies may benefit from incorporating clinical profiles to explore condition-specific differences in parent–provider dynamics.

While we recognize that retrospective designs may offer advantages in certain contexts, the use of a cross-sectional design was appropriate given this study’s aim to assess perceptions at a specific point in time and its feasibility within a clinical setting. Statistically, this study involved multiple group comparisons without correction for Type I error, which may increase the likelihood of spurious findings. Although efforts were made to ensure only one response per parent–child dyad, the risk of non-independence was considered and clarified during data handling. Finally, the use of self-administered questionnaires introduces the potential for response bias, including social desirability effects and subjective interpretation of item content.

### 4.2. Implication for the Nursing Practice

This study highlights the imperative need to address the needs of both parents and children, with particular attention to the psychological and emotional support required by parents during their child’s hospitalization. This provides an opportunity for nurses to enhance their practice by recognizing and addressing the emotional needs of parents, not just the medical needs of the child.

One key implication is the need for nurses to actively engage with parents in the care process, establishing trust and clear communication. As this study found that parents value trust and information, nurses must prioritize building a trusting relationship through transparent and empathetic communication. This could involve providing regular updates on the child’s condition, offering emotional support, and ensuring that parents feel involved in decision-making. Nurses should also be trained to recognize signs of parental anxiety and offer appropriate support or referrals to counseling services, if necessary [35]. Another implication is the need for targeted interventions to improve nurses’ ability to address both informational and emotional needs. This study suggests that parents felt the need for more detailed information than nurses perceived, which may indicate a gap in communication. Nurses can enhance their practice by improving their communication skills, ensuring that parents receive the information they need in a clear and timely manner [36]. Additionally, given the emotional strain that hospitalization places on families, interventions aimed at improving nurses’ competence in providing psychological support are critical. Incorporating these findings into nursing practice could lead to more holistic and family-centered care, ultimately improving the patient and family experience during hospitalization.

## 5. Conclusions

This study assessed and compared the perceptions of nurses and parents regarding the needs of families during pediatric hospitalization. While both parents and nurses rated key needs—such as trust, information, and emotional support—as highly important, differences emerged in the relative prioritization and perceived fulfillment of these needs. Parents consistently rated these needs as slightly more critical and reported greater concern over whether they were adequately met compared to nurses. These perceptual gaps suggest opportunities to improve mutual understanding and enhance communication practices to strengthen family-centered care. While some differences reached statistical significance at *p* < 0.01, others were only suggestive, highlighting areas for further exploration regarding perceived care delivery. These findings highlight the importance of understanding role-based differences in perception within pediatric care and underscore the relevance of communication and relational dynamics in family-centered practice. Further research is warranted to examine how such perceptual differences influence care processes and to identify strategies for fostering shared understanding between healthcare providers and families.

## Figures and Tables

**Table 1:** Sociodemographic characteristics of participants.

Characteristics of Parents		*n* (%)
Relation to the child	Mothers Fathers	199 (91.3%) 19 (8.7%)
Age of parents	Below 20 21–30 31–40 Above 40	5 (2.3%) 60 (27.5%) 107 (49.1%) 46 (21.1%)
Educational level	Primary Secondary University Postgraduate	54 (24.8%) 72 (33%) 87 (39.9%) 5 (2.3%)
Occupation	Working Not working	64 (29.4%) 154 (70.6%)
Age of children	Below 1 1–5 5–10 Above 10	36 (16.5%) 83 (38.1%) 61 (28%) 38 (17.4%)
Hospital admission of children	Previous admission Admission to the same hospital	137 (62.8%) 113 (51.8%)
Demographic Characteristics of Nurses		
Age	20–30 31–40 41–50 51–60	52 (23.9%) 112 (51.7%) 48 (22.1%) 5 (2.3%)
Nationality	Saudi Non-Saudi	76 (34.9%) 142 (65.1%)
Highest level of education	University degree Diploma Graduate certificate	114 (52.3%) 66 (30.3%) 37 (17%)
Years of experience	Mean (SD) Range	34 (2.78%) 1–18

**Table 2:** Comparison of parent and nurse NPQ mean scores by category: importance, fulfillment, and independence.

Category	Importance (P/S)	*p*	Fulfillment (P/S)	*p*	Independence (P/S)	*p*
Trusted (Parents’ trust in nurses/medical care)	3.97/3.91	0.01 *	5.45/4.68	<0.001 *	2.13/2.09	0.15
To be trusted (Parents feeling trusted)	7.86/7.90	0.29	10.76/11.07	0.02 *	4.21/4.16	0.27
Information (Communication and clarity)	19.59/19.67	0.29	27.96/28.08	0.60	10.58/10.29	0.02 *
Support and Guidance (Emotional/practical support)	23.2/23.4	0.18	31.9/32.4	0.16	13.1/12.7	0.03 *
Human and Physical Resources (Staff and facilities)	27.5/27.6	0.87	37.7/38.3	0.15	14.7/14.9	0.60
Family (Family-centered care needs)	17.34/17.51	0.09	24.15/24.08	0.82	9.95/10.06	0.43

Notes: P = parent score; S = nurse score; *p*-values = statistically significant (*p* < 0.05 *).

## Data Availability

The datasets for the current study are available from the corresponding author upon reasonable request for data security. The data are not publicly available due to containing information that could compromise privacy.

## Supplementary Materials

The following supporting information can be downloaded at: https://www.mdpi.com/article/10.3390/children12070947/s1, Table S1. Parent and Nurses NPQ Responses: Trusted. Table S2. Parent and Nurses NPQ Responses: To be Trusted. Table S3. Parent and Nurses NPQ Responses: Information. Table S4. Parent and Nurses NPQ Responses: support and guidance. Table S5. Parent and nurses NPQ Responses: Human and physical resources. Table S6. Parent and nurses NPQ Responses: family.
