# Supplementary material for: Perceptions of Parental Needs in General Pediatric Inpatient Units: A Comparative Study Between Nurses and Parents in Saudi Arabia
Source: Children (Basel). 2025 Jul 18;12(7):947. doi: 10.3390/children12070947 (PMC12293146; doi:10.3390/children12070947)
Supplement: Supplementary file 1 [file children-12-00947-s001.zip › children-3716452-supplementary.pdf]

Supplemental Table S1. Parent and Nurses NPQ Responses: Trusted.

Needs statements: parent (P), Nurses (S)	I *n* (%)	NI *n* (%)	*p*	FM *n* (%)	SM *n* (%)	NM *n* (%)	*p*	Y *n* (%)	N *n* (%)	*p*
4 To be sure that although am not present, my child will get the best available nursing care	P 215 (98.6) S 210 (96.3)	P 3 (1.4) S 8 (3.7)	0.28	P 164 (75.2) S 168 (77.1)	P 30 (13.8) S 33 (15.1)	P 24 (11) S 17 (7.8)	0.50	P 204 (93.6) S 207 (95)	P 14 (6.4) S 11 (5)	0.54
32 To be able to trust that although I am not present, my child will get the best available medical care	P 216 (99.1) S 206 (94.5)	P 2 (0.9) S 12 (5.5)	0.06	P 184 (84.4) S 180 (82.6)	P 14 (6.4) S 24 (11)	P 20 (9.2) S 14 (6.4)	0.16	P 200 (91.7) S 210 (96.3)	P 18 (8.3) S 8 (3.7)	0.09
Category A	Importance			Fulfilment				Independence		
Trust Mean Score (rank)	P 3.97	S 3.91	0.01	P 5.45	S 4.68		<0.001	P 2.13	S 2.09	0.15

Supplemental Table S2. Parent and Nurses NPQ Responses: To be Trusted

Needs statements: parent (P), Nurses (S)	I *n* (%)	NI *n* (%)	*p*	FM *n* (%)	SM *n* (%)	NM *n* (%)	*p*	Y *n* (%)	N *n* (%)	*p*
18 To feel that I am trusted to be able to care for my child in hospital	P 211 (96.8) S 214 (98.2)	P 7 (3.2) S 4 (1.8)	0.36	P 173 (79.4) S 188 (86.2)	P 25 (11.5) S 24 (11)	P 20 (9.2) S 6 (2.8)	0.02	P 210 (96.3) S 213 (97.7)	P 8 (3.7) S 5 (2.3)	0.40
26 To feel that I am not blamed for my child's illness	P 210 (96.3) S 207 (95)	P 8 (3.7) S 11 (5)	0.48	P 187 (85.8) S 178 (81.7)	P 15 (6.9) S 28 (12.8)	P 16 (7.3) S 12 (5.5)	0.09	P 200 (91.7) S 205 (94)	P 18 (8.3) S 13 (6)	0.35
34 That nurses contact and consult me about the care that is needed for the nursing care of my child	P 212 (97.2) S 216 (99.1)	P 6 (2.8) S 2 (0.9)	0.15	P 161 (73.9) S 178 (73.9)	P 23 (10.6) S 29 (13.3)	P 34 (15.6) S 11 (5)	0.01	P 213 (97.7) S 209 (95.9)	P 5 (2.3) S 9 (4.1)	0.28
40 To feel that I am needed in the ward/ unit	P 209 (95.9) S 213 (97.7)	P 9 (4.1) S 5 (2.3)	0.28	P 172 (78.9) S 166 (76.1)	P 25 (11.5) S 40 (18.3)	P 21 (9.6) S 12 (5.5)	0.06	P 203 (93.1) S 210 (96.3)	P 15 (6.9) S 8 (3.7)	0.13
Category B	Importance			Fulfilment				Independence		
Trusted Mean Score (rank)	P 7.86	S 7.90	0.29	P 10.76	S 11.07		0.02	P 4.21	S 4.16	0.27

Supplemental Table S3. Parent and Nurses NPQ Responses: Information.

Needs statements: parent (P), nurses (S)	I *n* (%)	NI *n* (%)	*p*	FM *n* (%)	SM *n* (%)	NM *n* (%)	*p*	Y *n* (%)	N *n* (%)	*p*
6 To be able to see a social worker to get information about financial assistance to help ease problems	P 174 (79.8) S 198 (90.8)	P 44 (20.2) S 20 (9.2)	0.01	P 144 (66.4) S 173 (79.4)	P 26 (12) S 20 (9.2)	P 47 (21.7) S 25 (11.5)	0.01	P 180 (82.6) S 208 (95.4)	P 38 (17.4) S 10 (4.6)	<0.0
8 That I receive written information about my child's health status so I can review it later	P 211 (96.8) S 217 (99.5)	P 7 (3.2) S 1 (0.5)	0.03	P 167 (76.6) S 180 (82.6)	P 23 (10.6) S 22 (10.1)	P 28 (12.8) S 16 (7.3)	0.15	P 201 (92.2) S 208 (95.4)	P 17 (7.8) S 10 (4.6)	0.16
15 That I be informed about all known health outcomes for my child	P 216 (99.1) S 215 (98.6)	P 2 (0.9) S 3 (0.9)	0.65	P 202 (92.7) S 200 (91.7)	P 12 (5.5) S 13 (0.6)	P 4 (1.8) S 5 (2.3)	0.92	P 213 (97.7) S 212 (97.2)	P 5 (2.3) S 6 (2.8)	0.76
19 That I be informed about all treatment that my child will receive	P 216 (99.1) S 211 (96.8)	P 2 (0.9) S 7 (3.2)	0.09	P 202 (92.7) S 182 (83.5)	P 14 (6.4) S 21 (9.6)	P 2 (0.9) S 15 (6.9)	0.02	P 214 (98.2) S 211 (96.8)	P 4 (1.8) S 7 (3.2)	0.36
24 To learn and be informed about how illness affects children's growth and development	P 210 (96.3) S 216 (99.1)	P 8 (3.7) S 2 (0.9)	0.06	P 193 (88.5) S 193 (88.5)	P 16 (7.3) S 16 (7.3)	P 9 (4.1) S 9 (4.1)	1.0	P 205 (94) S 210 (96.3)	P 13 (6) S 8 (3.7)	0.26
29 That I be prepared for the day of discharge and any change in that date	P 212 (97.2) S 212 (97.2)	P 6 (2.8) S 6 (2.8)	1.0	P 197 (90.4) S 191 (87.6)	P 10 (4.6) S 14 (6.4)	P 11 (5) S 13 (6)	0.63	P 214 (98.2) S 215 (98.6)	P 4 (1.8) S 3 (1.4)	0.70

31 That I be informed as soon as possible about results from tests done	P 214 (98.2) S 210 (96.3)	P 4 (1.8) S 8 (3.7)	0.24	P 198 (90.8) S 190 (87.2)	P 11 (5) S 18 (8.3)	P 9 (4.1) S 10 (4.6)	0.39	218 (100) 218 (100)	- -	NA
38 That I get exact information about my child's condition	P 217 (99.5) S 214 (98.2)	P 1 (0.5) S 4 (1.8)	0.18	P 192 (88.1) S 201 (92.2)	P 12 (5.5) S 11 (5)	P 14 (6.4) S 6 (2.8)	0.18	P 206 (94.5) S 213 (97.7)	P 12 (5.5) S 5 (2.3)	0.08
43 To be told about everything that is being done to or for my child and why	P 216 (99.1) S 216 (99.1)	P 2 (0.9) S 2 (0.9)	1.0	P 205 (94) S 199 (91.3)	P 10 (4.6) S 15 (6.9)	P 3 (1.4) S 4 (1.8)	0.54	P 210 (96.3) S 213 (97.7)	P 8 (3.7) S 5 (2.3)	0.40
49 That one person (a nurse) coordinates the services and flow of information we get in hospital	P 204 (93.6) S 198 (90.8)	P 14 (6.4) S 20 (9.2)	0.28	P 173 (79.4) S 170 (78)	P 28 (12.8) S 34 (15.6)	P 17 (7.8) S 14 (6.4)	0.64	P 193 (88.5) S 208 (95.4)	P 25 (11.5) S 10 (4.6)	0.01
Category C	Importance			Fulfilment				Independence		
Information Mean Score (rank)	P 19.59	S 19.67	0.29	P 27.96	S 28.08		0.60	P 10.58	S 10.29	0.02

Supplemental Table S4. Parent and Nurses NPQ Responses: support and guidance.

Needs statements: parent (P), nurses (S)	I *n* (%)	NI *n* (%)	*p*	FM *n* (%)	SM *n* (%)	NM *n* (%)	*p*	Y *n* (%)	N *n* (%)	*p*
2 To have a planned meeting with other parents to share and discuss experience of my child's Hospitalization	P 183 (83.9) S 198 (90.8)	P 35 (16.1) S 20 (9.2)	0.03	P 138 (63.3) S 151 (69.3)	P 29 (13.3) S 28 (12.8)	P 51 (23.4) S 39 (17.9)	0.33	P 185 (84.9) S 195 (89.4)	P 33 (15.1) S 23 (10.6)	0.15
3 That nurses encourage parents to ask questions and	P 211 (96.8) S 212 (97.2)	P 7 (3.2) S 6 (2.8)	0.78	P 175 (80.3) S 171 (78.4)	P 26 (11.9) S 39 (17.9)	P 17 (7.8) S 8 (3.7)	0.01	P 209 (95.9)S 210 (96.3)	P 9 (4.1) S 8 (3.7)	0.81

seek answers to them										
7 To be able to meet with parents with similar experiences of an ill child	P 175 (80.3) S 188 (86.2)	P 43 (19.7) S 30 (13.8)	0.10	P 126 (57.8) S 138 (63.3)	P 20 (9.2) S 30 (13.8)	P 72 (33) S 50 (22.9)	0.04	P 163 (74.8) S 194 (89)	P 55 (25.2) S 24 (11)	0.00
9 To be able to ask nurses and doctors about how to explain the illness and/or tests to my child	P 217 (99.5) S 214 (98.2)	P 1 (0.5) S 4 (1.8)	0.18	P 189 (86.7) S 186 (85.3)	P 24 (11) S 22 (10.1)	P 5 (2.3) S 10 (4.6)	0.41	P 209 (95.9)S 215 (98.6)	P 9 (4.1) S 3 (1.4)	0.08
11 To have a person in the unit (a nurse or a doctor) especially assigned to respond to parents' needs	P 209 (95.9) S 207 (95)	P 9 (4.1) S 11 (5)	0.65	P 159 (72.9)S 152 (69.7)	P 27 (12.4) S 39 (17.9)	P 32 (14.7) S 27 (12.4)	0.25	P 202 (92.7)S 203 (93.1)	P 16 (7.3) S 15 (6.9)	0.85
13 That I get advice about the care of my child in preparation for my child's discharge	P 214 (98.2) S 215 (98.6)	P 4 (1.8) S 3 (1.4)	0.70	P 199 (91.3)S 192 (88.1)	P 10 (4.6) S 18 (8.3)	P 9 (4.1) S 8 (3.7)	0.29	P 207 (95) S 211 (96.8)	P 11 (5) S 7 (3.2)	0.34
16 To be encouraged by nurses to come and stay with my child from tests done	P 207 (95) S 203 (93.1)	P 11 (5) S 15 (6.9)	0.42	P 191 (87.6)S 185 (84.9)	P 17 (7.8) S 20 (9.2)	P 10 (4.6) S 13 (6)	0.69	P 199 (91.3)S 213 (97.7)	P 19 (8.7) S 5 (2.3)	0.03
17 That a nurse assists me to recognize my own needs,	P 194 (89) S 208 (95.4)	P 24 (11) S 10 (4.6)	0.01	P 153 (70.2) S 157 (72)	P 26 (11.9)S 43 (19.7)	P 39 (17.9) S 18 (8.3)	0.03	P 201 (92.2)S 207 (95)	P 17 (7.8) S 11 (5)	0.24

e.g. meals, sleep									
36 To know that I can contact the ward/ unit after my child has been discharged	P 193 (88.5) S 188 (86.2)	P 25 (11.5) S 30 (13.8)	0.47	P 152 (69.7)S 155 (71.1)	P 16 (7.3) S 22 (10.1)	P 50 (22.9) S 41 (18.8)	0.39	P 186 (85.3 S 184 (84.4)	P 32 (14.7) S 34 (15.6)	0.79
42 That I get assistance to recognize the needs of my child	P 216 (99.1) S 216 (98.1)	P 2 (0.9) S 2 (0.9)	1.0	P 197 (90.4)S 198 (90.8)	P 18 (8.3) S 17 (7.8)	P 3 (1.4) S 3 (1.4)	0.99	P 214 (98.2)S 215 (98.6)	P 4 (1.8) S 3 (1.4)	0.70
44 That I can continue to feel hopeful about my child's Condition	P 216 (99.1) S 213 (97.7)	P 2 (0.9) S 5 (2.3)	0.25	P 189 (86.7)S 193 (88.5)	P 19 (8.7) S 24 (11)	P 10 (4.6) S 1 (0.5)	0.02	P 205 (94) S 212 (97.2)	P 13 (6) S 6 (2.8)	0.10
50 That I do not feel hopeless	P 211 (96.8) S 215 (98.6)	P 7 (3.2) S 3 (1.4)	0.20	P 174 (79.8) S 183 (83.9)	P 25 (11.5)S 30 (13.8)	P 19 (8.7) S 5 (2.3)	0.01	200 (91.7)204 (93.6)	18 (8.3) 14 (6.4)	0.46
Category D	Importance			Fulfilment				Independence		
Support Mean Score (rank)	P 23.2	S 23.4	0.18	P 31.9	S 32.4		0.16	P 13.1	S 12.7	0.03

Supplemental Table S5. Parent and nurses NPQ Responses: Human and physical resources.

Needs statements: parent (P), nurses (S)	I *n* (%)	NI *n* (%)	*p*	FM *n* (%)	SM *n* (%)	NM *n* (%)	*p*	Y *n* (%)	N *n* (%)	*p*
1 To have a special place in the unit where parents can be by themselves	P 204 (93.6) S 183 (83.9)	P 14 (6.4) S 35 (16.1)	0.00	P 139 (63.8) S 117 (53.7)	P 35 (16.1) S 43 (19.7)	P 44 (20.2) S 58 (26.6)	0.09	P 194 (89) S 195 (89.4)	P 24 (11) S 23 (10.6)	0.88
5 That I get sufficient rest or adequate sleep	P 203 (93.1) S 217 (99.5)	P 15 (6.9) S 1 (0.5)	0.00	P 139 (63.8) S 160 (73.4)	P 41 (18.8) S 48 (22)	P 38 (17.4) S 10 (4.6)	0.00	P 211 (96.8) S 208 (95.4)	P 7 (3.2) S 10 (4.6)	0.46
10 That there is flexibility in the work of the unit according to parents' needs	P 214 (98.2) S 214 (98.2)	P 4 (1.8) S 4 (1.8)	1.0	P 175 (80.3) S 160 (73.4)	P 25 (11.5) S 54 (24.8)	P 18 (8.3) S 4 (1.8)	0.00	P 207 (95) S 212 (97.2)	P 11 (5) S 6 (2.8)	0.22

12 That I get an opportunity to speak privately with a doctor or a nurse about my own feelings or worries	P 209<br>(95.9)<br>S 216<br>(99.1)	P 9<br>(4.1)<br>S 2<br>(0.9)	0.03	P 180<br>(82.6)<br>S 184<br>(84.4)	P 21<br>(9.6)<br>S 28<br>(12.8)	P 17<br>(7.8)<br>S 6<br>(2.8)	0.04	P 212<br>(97.2)<br>S 215<br>(98.6)	P 6<br>(2.8)<br>S 3<br>(1.4)	0.31
14 That I be permitted to make the final decision about the treatment my child will receive	P 217<br>(99.5)<br>S 216<br>(99.1)	P 1<br>(0.5)<br>S 2<br>(0.9)	0.56	P 194<br>(89)<br>S 184<br>(84.4)	P 16<br>(7.3)<br>S 26<br>(11.9)	P 8<br>(3.7)<br>S 8<br>(3.7)	0.27	P 212<br>(97.2)<br>S 215<br>(98.6)	P 6<br>(2.8)<br>S 3<br>(1.4)	0.31
21 That I have a place to sleep in the hospital	P 211<br>(96.8)<br>S 212<br>(97.2)	P 7<br>(3.2)<br>S 6<br>(2.8)	0.78	P 169<br>(77.5)<br>S 164<br>(75.2)	P 23<br>(10.6)<br>S 44<br>(20.2)	P 26<br>(11.9)<br>S 10<br>(4.6)	0.00	P 210<br>(96.3)<br>S 208<br>(95.4)	P 8<br>(3.7)<br>S 10<br>(4.6)	0.63
33 That nurses recognize and understand the feelings of parents	P 201<br>(92.2)<br>S 211<br>(96.8)	P 17<br>(7.8)<br>S 7<br>(3.2)	0.04	P 178<br>(81.7)<br>S 182<br>(83.5)	P 15<br>(6.9)<br>S 20<br>(9.2)	P 25<br>(11.5)<br>S 16<br>(7.3)	0.26	P 197<br>(90.4)<br>S 205<br>(94)	P 21<br>(9.6)<br>S 13<br>(6)	0.15
35 To feel that I am important in contributing to my child's wellbeing	P 218<br>(100)<br>S 214<br>(98.2)	P 0<br>S 4<br>(1.8)	0.05	P 185<br>(84.9)<br>S 191<br>(87.6)	P 19<br>(8.7)<br>S 20<br>(9.2)	P 14<br>(6.4)<br>S 7<br>(3.2)	0.29	P 209<br>(95.9)<br>S 206<br>(94.5)	P 9<br>(4.1)<br>S 12<br>(5.5)	0.50
37 That I get assistance and support to recognize and understand my own needs, e.g. anxiety, tiredness	P 209<br>(95.9)<br>S 212<br>(97.2)	P 9<br>(4.1)<br>S 6<br>(2.8)	0.43	P 169<br>(77.5)<br>S 182<br>(83.5)	P 18<br>(8.3)<br>S 15<br>(6.9)	P 31<br>(14.2)<br>S 21<br>(9.6)	0.26	P 204<br>(93.6)<br>S 194<br>(89)	P 14<br>(6.4)<br>S 24<br>(11)	0.09
39 That I feel less anxious	P 212<br>(97.2)<br>S 211<br>(96.8)	P 6<br>(2.8)<br>S 7<br>(3.2)	0.78	P 177<br>(81.2)<br>S 194<br>(89)	P 20<br>(9.2)<br>S 19<br>(8.7)	P 21<br>(9.6)<br>S 5<br>(2.3)	0.00	P 214<br>(98.2)<br>S 210<br>(96.3)	P 4<br>(1.8)<br>S 8<br>(3.7)	0.24
41 To be able to ‘room in’ with my child	P 216<br>(99.1)<br>S 218<br>(100)	P 2<br>(0.9)<br>S 0	0.16	P 200<br>(91.7)<br>S 193<br>(88.5)	P 9<br>(4.1)<br>S 20<br>(9.2)	P 9<br>(4.1)<br>S 5<br>(2.3)	0.07	P 210<br>(96.3)<br>S 214<br>(98.2)	P 8<br>(3.7)<br>S 4<br>(1.8)	0.24

45 That I can have meals with my child on the ward/unit	P 217 (99.5) S 214 (98.2)	P 1 (0.5) S 4 (1.8)	0.18	P 192 (88.1) S 202 (92.7)	P 21 (9.6) S 8 (3.7)	P 5 (2.3) S 8 (3.7)	0.03	P 212 (97.2) S 212 (97.2)	P 6 (2.8) S 6 (2.8)	1.0
46 That there are bath and shower facilities for parents	P 208 (95.4) S 207 (95)	P 10 (4.6) S 11 (5)	0.82	P 167 (76.6) S 170 (78)	P 22 (10.1) S 23 (10.6)	P 29 (13.3) S 25 (11.5)	0.84	P 204 (93.6) S 188 (86.2)	P 14 (6.4) S 30 (13.8)	0.01
47 To know that my child will get proper schooling so he/she will not fall behind in development	P 204 (93.6) S 201 (92.2)	P 14 (6.4) S 17 (7.8)	0.58	P 165 (75.7) S 161 (73.9)	P 21 (9.6) S 38 (17.4)	P 32 (14.7) S 19 (8.7)	0.02	P 198 (90.8) S 198 (90.8)	P 20 (9.2) S 20 (9.2)	1.0
Category E	Importance			Fulfilment				Independence		
Human resources Mean Score (rank)	P 27.5	S 27.6	0.87	P 37.7	S 38.3		0.15	P 14.7	S 14.9	0.60

Supplemental Table S6. Parent and nurses NPQ Responses: family.

Needs statements: parent (P), nurses (S)	I *n* (%)	NI *n* (%)	*p*	FM *n* (%)	SM *n* (%)	NM *n* (%)	*p*	Y *n* (%)	N *n* (%)	*p*
20 To have a person in the unit especially assigned to take care of the needs of my child	P 202 (92.7) S 212 (97.2)	P 16 (7.3) S 6 (2.8)	0.03	P 176 (80.7) S 167 (76.6)	P 18 (8.3) S 38 (17.4)	P 24 (11) S 13 (6)	0.01	P 207 (95) S 203 (93.1)	P 11 (5) S 15 (6.9)	0.42
22 That a nurse follows up my child after discharge	P 195 (89.4) S 195 (89.4)	P 23 (10.6) S 23 (10.6)	1.0	P 137 (62.8) S 173 (62.8)	P 22 (10.1) S 33 (15.1)	P 59 (27.1) S 48 (22)	0.19	P 183 (83.9) S 169 (77.5)	P 35 (16.1) S 49 (22.5)	0.09
23 To be able to participate in the nursing care of my child	P 213 (97.7) S 214 (98.2)	P 5 (2.3) S 4 (1.8)	0.74	P 192 (88.1) S 186 (85.3)	P 11 (5) S 24 (11)	P 15 (6.9) S 8 (3.7)	0.03	P 209 (95.9) S 215 (98.6)	P 9 (4.1) S 3 (1.4)	0.08
25 That I can stay with my child 24 h a day if I wish	P 218 (100) S 217 (99.5)	P 0 S 1 (0.5)	0.32	P 200 (91.7) S 191 (87.6)	P 14 (6.4) S 22 (10.1)	P 4 (1.8) S 5 (2.3)	0.35	P 215 (98.6) S 205 (94)	P 3 (1.4) S 13 (6)	0.01

27 To be able to do physical care for my child, e.g. change nappy, bath, feed, etc.	P 204 (93.6)<br>S 202 (92.7)	P 14 (6.4)<br>S 16 (7.3)	0.71	P 193 (88.5)<br>S 188 (86.2)	P 10 (4.6)<br>S 10 (4.6)	P 15 (6.9)<br>S 20 (9.2)	0.68	P 206 (94.5)<br>S 208 (95.4)	P 12 (5.5)<br>S 10 (4.6)	0.66
28 That I be able to explain things to my relations, friends, and my other child/children	P 204 (93.6)<br>S 210 (96.3)	P 14 (6.4)<br>S 8 (3.7)	0.19	P 188 (86.2)<br>S 166 (76.1)	P 19 (8.7)<br>S 40 (18.3)	P 11 (5)<br>S 12 (5.5)	0.01	P 196 (89.9)<br>S 197 (90.4)	P 22 (10.1)<br>S 21 (9.6)	0.87
30 That I have time to be with my other child/ children	P 207 (95)<br>S 200 (91.7)	P 11 (5)<br>S 18 (8.3)	0.18	P 168 (77.1)<br>S 162 (74.3)	P 21 (9.6)<br>S 33 (15.1)	P 29 (13.3)<br>S 23 (10.6)	0.18	P 200 (91.7)<br>S 196 (89.9)	P 18 (8.3)<br>S 22 (10.1)	0.51
48 That the same nurses take care of my child most of the time	P 201 (92.2)<br>S 210 (96.3)	P 17 (7.8)<br>S 8 (3.7)	0.06	P 174 (79.8)<br>S 156 (71.6)	P 20 (9.2)<br>S 54 (24.8)	P 24 (11)<br>S 8 (3.7)	0.00	P 197 (90.4)<br>S 192 (88.1)	P 21 (9.6)<br>S 26 (11.9)	0.44
51 That qualified teachers are available to ensure that my child's development is maintained	P 174 (79.8)<br>S 196 (89.9)	P 44 (20.2)<br>S 22 (10.1)	0.03	P 146 (67.3)<br>S 150 (68.8)	P 21 (9.7)<br>S 28 (12.8)	P 40 (18.3)<br>S 50 (23)	0.34	P 142 (65.1)<br>S 147 (67.4)	P 76 (34.9)<br>S 71 (32.6)	0.61
Category F	Importance			Fulfilment				Independence		
Family members Mean Score (rank)	P 17.34	S 17.51	0.09	P 24.15	S 24.08		0.82	P 9.95	S 10.06	0.43
